# Geographic Distribution of Radiologists and Utilization of Teleradiology in Japan: A Longitudinal Analysis Based on National Census Data

**DOI:** 10.1371/journal.pone.0139723

**Published:** 2015-09-30

**Authors:** Masatoshi Matsumoto, Soichi Koike, Saori Kashima, Kazuo Awai

**Affiliations:** 1 Department of Community-Based Medical System, Faculty of Medicine, Hiroshima University, Hiroshima, Japan; 2 Division of Health Policy and Management, Center for Community Medicine, Jichi Medical University, Tochigi, Japan; 3 Department of Public Health and Health Policy, Institute of Biomedical and Health Sciences, Hiroshima University, Hiroshima, Japan; 4 Department of Diagnostic Radiology, Institute of Biomedical and Health Sciences, Hiroshima University, Hiroshima, Japan; Hamamatsu University School of Medicine, JAPAN

## Abstract

**Background:**

Japan has the most CT and MRI scanners per unit population in the world, and as these technologies spread, their geographic distribution is becoming equalized. In contrast, the number of radiologists per unit population in Japan is the lowest among OECD countries and their geographic distribution is unknown. Likewise, little is known about the use of teleradiology, which can compensate for the uneven distribution of radiologists.

**Methods:**

Based on the Survey of Physicians, Dentists and Pharmacists and the Static Survey of Medical Institutions by the Ministry of Health, Labour and Welfare, a dataset of radiologists and CT and MRI utilizations in each of Japan’s 1811 municipalities was created. The inter-municipality equity of the number of radiologists was evaluated using Gini coefficient. Logistic regression analysis, based on Static Survey data, was performed to evaluate the association between hospital location and teleradiology use.

**Results:**

Between 2006 and 2012 the number of radiologists increased by 21.7%, but the Gini coefficient remained unchanged. The number of radiologists per 1,000 CT (MRI) utilizations decreased by 17.9% (1.0%); the number was highest in metropolis and lowest in town/village and the disparity has widened from 1.9 to 2.2 (1.6 to 2.0) times. The number of hospitals and clinics using teleradiology has increased (by 69.6% and 18.1%, respectively). Hospitals located in towns/villages (odds ratio 1.61; 95% confidence interval 1.26–2.07) were more likely to use teleradiology than those in metropolises.

**Conclusions:**

Contrary to the CT and MRI distributions, radiologist distribution has not been evened out by the increase in their number; in other words, the distribution of radiologists was not affected by market-derived spatial competition force. As a consequence, the gap of the radiologist shortage between urban and rural areas is increasing. Teleradiology, which is one way to ameliorate this gap, should be encouraged.

## Introduction

Japan has the most computed tomography (CT) and magnetic resonance imaging (MRI) scanners per unit population in the world [1]. In a previous study, we reported the geographic distribution of these diagnostic modalities across Japan [2]. We showed that the more abundant a modality, the more equal its distribution, and any increase in the modality makes its distribution even more equal. This suggests that the geographic distribution of the diagnostic imaging technology in Japan confirms the spatial competition hypothesis [2]. The spatial competition hypothesis is an economic model in which service suppliers are affected by the maximization of profit and an increase in the supplier density will increase competition for profit in urban areas, which leads suppliers to rural areas to improve the equity of physician distribution [3,4].

In contrast the relationship between the number and the geographic distribution of radiologists is unknown. Despite the large number of imaging devices, the number of radiologists per unit population in Japan is the lowest among OECD countries [5], and is less than half of the number in the United States [6]. Fewer radiologists are working with many more imaging devices in Japan than in other countries. In order to make the best use of limited human resources, it is necessary to reduce the gap between the geographic distribution of radiologists and that of imaging devices. In Japan the number of major imaging devices such as CT, MRI and positron-emission tomography (PET) is rapidly increasing, and their distribution is becoming evenly spread among municipalities [2]. The number of radiologists must be better matched to the distribution of these devices.

In reality, however, human resources such as physicians do not necessarily follow the spatial competition hypothesis [7–9]. It is possible that there is a substantial and widening gap between the distribution of imaging devices and that of radiologists despite the lack of scientific evidence showing it [10]. As a practical solution, teleradiology has been used since the 1990s [11]. Several trials have connected urban and rural hospitals and supported the rural hospitals’ use of teleradiology [11–13]. However there is scanty evidence of the nationwide status of teleradiology utilization in Japan.

In this study, based on national census data, we investigate the number and geographic distribution of radiologists in Japan and examine whether the number-distribution relationship is consistent with the spatial competition hypothesis. We also show the trend in the gap between the distribution of radiologists and of imaging devices. We then illustrate the current status and recent trend of teleradiology use among Japanese medical institutions. Based on the results, we predict a problem in radiology service provision and suggest a desirable policy option.

## Materials and Methods

### Radiologist data

Unpublicized individual data of the Survey of Physicians, Dentists and Pharmacists conducted in 2006, 2008, 2010 and 2012 were used. Permission to use the data for research was obtained from the Ministry of Health, Labour and Welfare. All licensed physicians in Japan are obligated to register in the Survey. Among registered physicians, only those whose main specialty was radiology were recognized as radiologists in this study.

The total number of physicians and that of radiologists in each municipality were calculated from the Survey. Because many municipalities were merged between 2006 and 2012, the municipality border in 2012 was applied to 2006, 2008 and 2010, and the number of municipalities was fixed in all years at 1811. According to the device data, municipalities affected by the Great East Japan Earthquake were deleted.

### Device data

Details of device data have been reported in a previous study [2]. Unpublicized individual data of the Static Survey of Medical Institutions in 2005, 2008 and 2011, which was conducted by the Ministry of Health, Labour and Welfare and covered all the hospitals and clinics in Japan, were used to create the dataset on the number of utilizations of CT and MRI scanners in each municipality. In the same manner, the dataset on the number of medical institutions using teleradiology in each municipality in each year was created. The 2011 survey did not cover all of the facilities in Fukushima and some of the facilities in the Miyagi prefecture because of the Great East Japan Earthquake. For this reason, data in these areas was deleted. The municipality border in each year was incorporated into that of 2012 as was radiologist data.

### Municipality data

The municipality population in 2012 was extracted from the Statistical Observations of Shi Ku, Machi, Mura 2013, published by the Statistics Bureau, Ministry of Internal Affairs and Communications[14]. The radiologist data and device data were connected to this municipality-based population data through the municipality code.

### Classification of municipality

The means of classifying municipalities was reported previously [2]. Japan has three levels of government: national, prefectural and municipal. A municipality consists of three types of community: city, town and village. In this study, municipalities were classified as “metropolis,” “city” and “town/village.” “Metropolis” includes all of the wards (*ku*) of the ordinance-designated cities (*seirei-shitei-toshi*) and 23 special wards of Tokyo (n = 171). “City” includes the other cities (*shi*) (n = 756). “Town/village” includes towns (*cho*) and villages (*son*) (n = 884). The number of metropolises and cities is slightly different from those in our previous study [2], because the link between different year datasets was done through postcodes in the previous study but through municipality codes in this study.

### Statistical analysis

The number of radiologists per 100,000 population in each municipality category (metropolis, city and town/village) was calculated using the data of the total number of radiologists and the total population in the category of municipalities. The number of radiologists per 1,000 CT in each category in each year was then calculated. Because years of radiologist data (2006, 2008, 2010 and 2012) do not agree with years of device data (2005, 2008 and 2011), 2006 radiologist data was applied to 2005 device data, and 2012 radiologist data was applied to 2011 device data. In a similar way, the number of radiologists per 1,000 MRI utilizations in each category in each year was calculated. The proportion of hospitals (or clinics) using teleradiology among all hospitals (or clinics) was also calculated.

To evaluate the inter-municipality equity of the number of devices per unit population, the Gini coefficient was calculated [2]. The Gini coefficient is used extensively in health-related literature to evaluate the inter-community equity of resources [7,15–21]. In the calculation of the Gini coefficient, all 1811 municipalities were ranked by the number of radiologists per 100,000 population. Each municipality was plotted onto the plane of coordinates with its x-axis being the cumulative proportion of the population and the y-axis being the cumulative proportion of radiologists. The plotted line is the Lorenz curve; the Gini coefficient is the area between the Lorenz curve and the 45-degree line, which is divided by the triangle under the 45-degree line. The Gini coefficient ranges from 0 (complete equity) to 1 (complete inequity), according to the variation in the number of radiologists per 100,000 population among the municipalities. A significance test was conducted to examine the difference in the Gini coefficient between years. This was accomplished by calculating the bootstrapped standard errors for the Gini coefficient.

Multivariate logistic regression analysis, based on the 2011 Survey data, was conducted to examine the characteristics of hospitals or clinics that use teleradiology. Data on all the individual hospitals (n = 8,632) and clinics (n = 101,083) was included in the analysis. The outcome variable was whether teleradiology was used. Explanatory variables in the multivariate regression model were location of the institution (metropolis, city or town/village), its ownership (private or public), whether or not it has a diagnostic device (CT, MRI or PET), the number of radiologists, and the number of inpatient beds. The odds ratio and its 95% confidence interval (95% CI) of each explanatory variable adjusted for other variables were calculated.

Spearman’s rank correlation coefficient was calculated to evaluate the strength of correlation between total population in each municipality and the number of radiologists per 100,000 population in each municipality. Similarly, the correlation coefficient was calculated between total population and the number of radiologists per 1,000 CT utilizations, and that between total population and the number of radiologists per 1,000 MRI utilizations in each municipality.

All but one of these statistical analyses were conducted using SPSS version 21 (IBM-SPSS Japan, Tokyo); the calculation of the Gini coefficients and the significance test for their differences were conducted with STATA software (version 12, College Station, TX, USA). All the maps shown in the Results were created using ArcGIS version 10.1 (ESRI Japan Inc.).

### Ethics Statement

The Ethics Committee of the Graduate School of Medicine and Faculty of Medicine at the University of Tokyo have both assessed and given permission for this study (Assessment Number 10493). The Ethics Committee for Epidemiological Research at Hiroshima University agreed to this permission and approved this study (assessment number 838).

## Results

**Fig 1:**
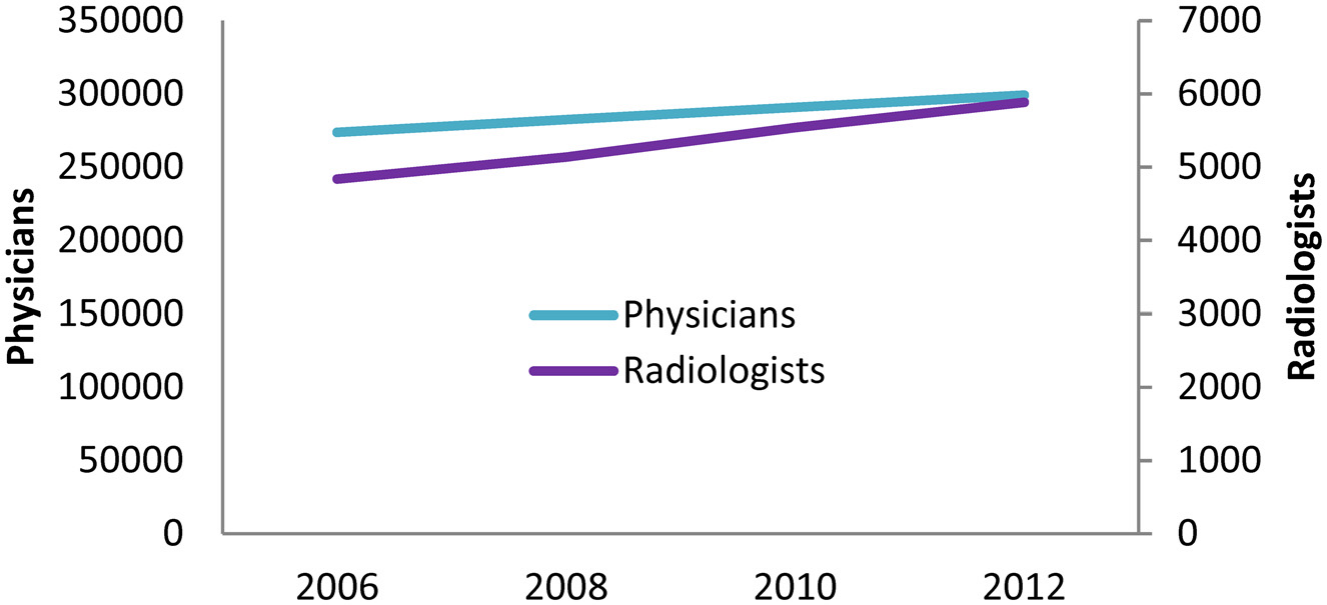
Number of physicians and radiologists in Japan.

Transition of the number of all physicians and that of radiologists is shown in Fig 1. Between 2006 and 2012, the number of physicians increased by 9.3% from 273,686 to 299,155. In the same period, the number of radiologists increased by 21.7% from 4,836 to 5,883, which exceeded the increased rate in the number of all physicians.

**Fig 2:**
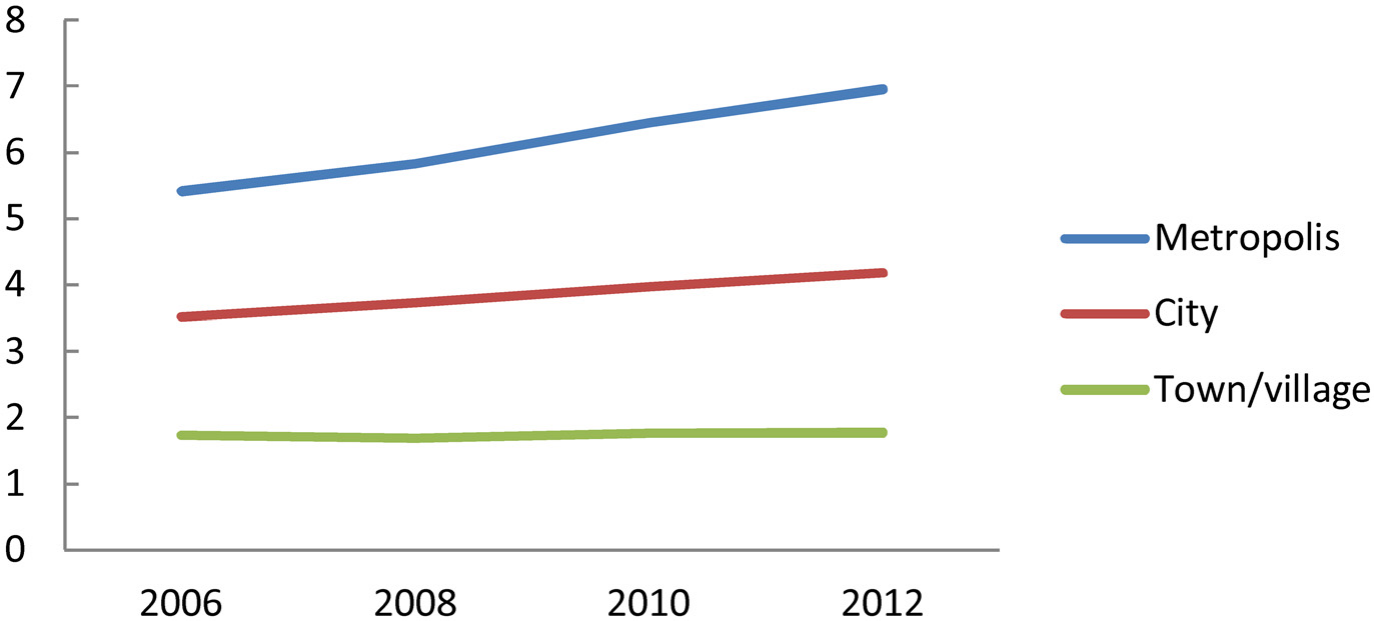
Number of radiologists per 100,000 population, classified by municipality type. “Metropolis” includes all the wards (*ku*) of the ordinance-designated cities (*seirei-shitei-toshi*), as well as 23 special wards of Tokyo (n = 171). “City” includes the other cities (*shi*) (n = 756); “town/village” includes towns (*cho*) and villages (*son*) (n = 884).

Transition of the number of radiologists per 100,000 population classified by municipality type is shown in Fig 2. The number of radiologists per 100,000 population was highest in metropolis and lowest in town/village. The gap of the value between metropolis and town/village was 3.1 times in 2006. Between 2006 and 2012, the value in metropolis increased by 28.4%, while that in town/village increased by 2.6%. Thus, the gap between metropolis and town/village increased by 3.9 times.

**Fig 3:**
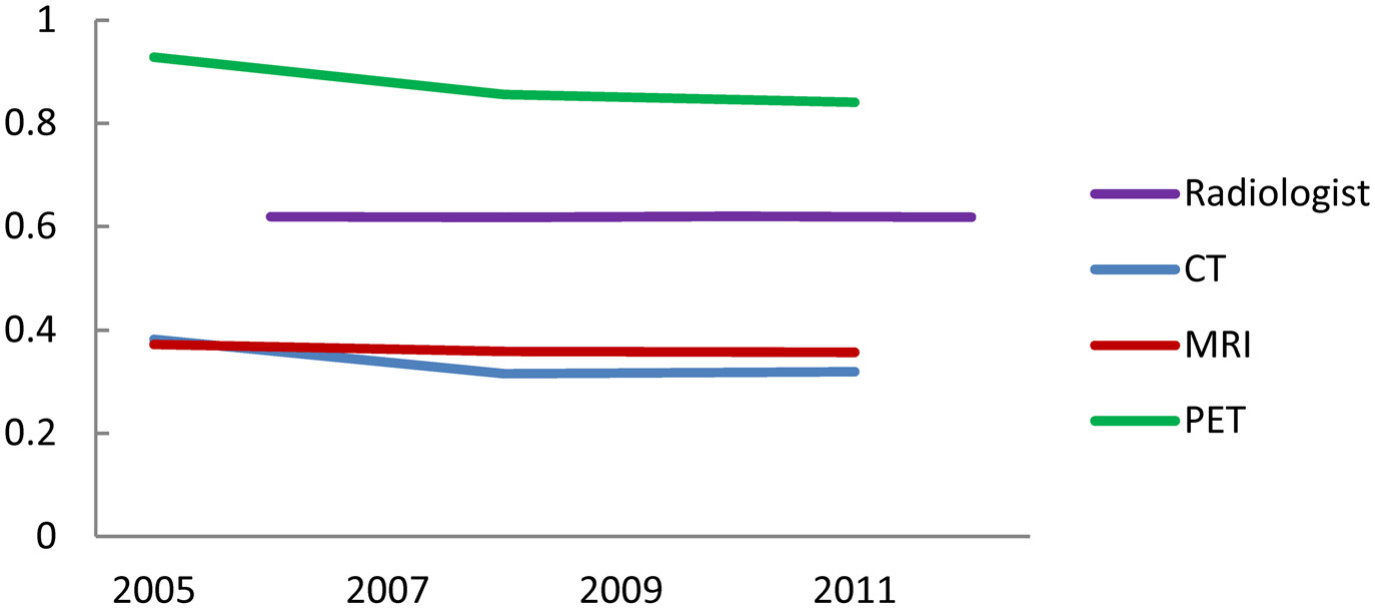
Gini coefficient of the number of radiologists and diagnostic device utilizations. Gini coefficients of diagnostic devices were excerpted from a previous study [2].

The Gini coefficient, an indicator of inter-municipality equity, of the number of radiologists is shown in Table 1. Lorenz curves are shown as supporting information in S1 Fig In spite of the rapid increase in the number of radiologists nationwide, Gini coefficient has remained almost unchanged between 2006 and 2012. As shown in Fig 3, this is in contrast to the transition of Gini coefficient of CT, MRI and PET, which significantly decreased in the similar period [2].

**Table 1:** Gini coefficient of the number of radiologists among municipalities.

	2006	2008	2010	2012
Gini	0.61912	0.61769	0.61961	0.61791
P for difference from 2006 value		0.754	0.927	0.83

**Fig 4:**
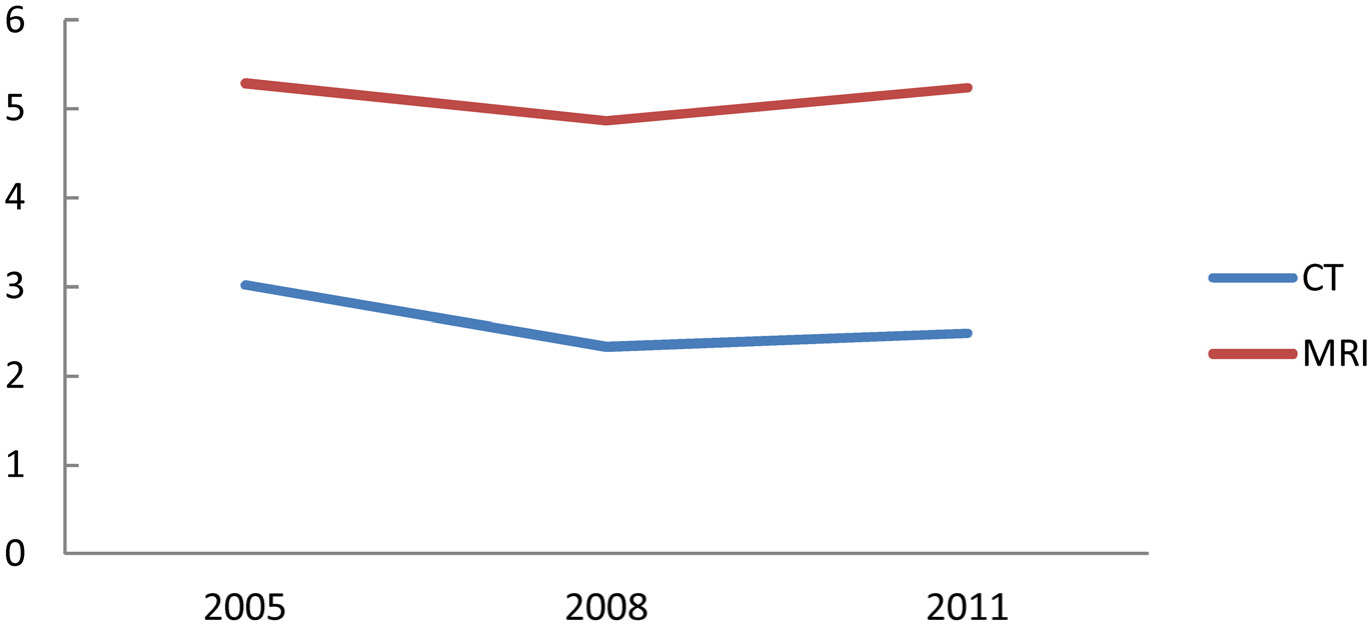
Number of radiologists per 1,000 CT and MRI utilizations.

The number of radiologists per 1,000 CT or MRI utilizations is shown in Fig 4. The number of radiologists per 1,000 CT utilizations has decreased by 17.7% for the six years, and that per 1,000 MRI utilizations has decreased by 1.0%. This suggests that the workload of radiologists increased in terms of CT.

**Fig 5:**
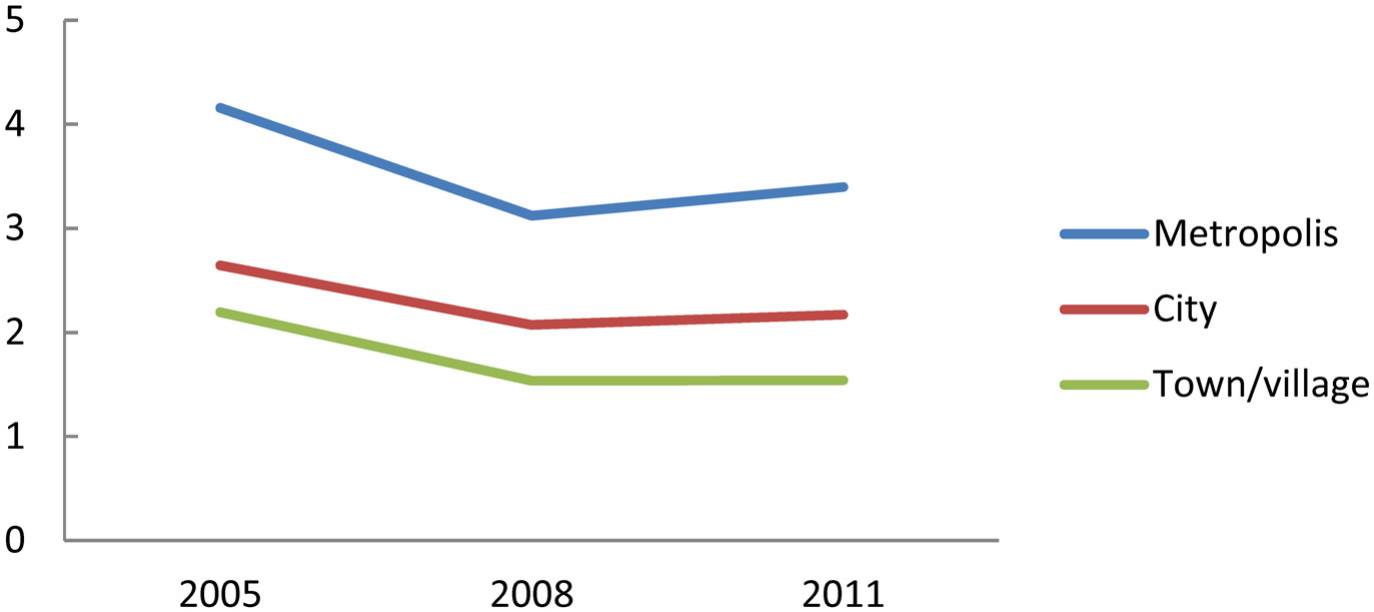
Number of radiologists per 1,000 CT utilizations, classified by municipality type.

The number of radiologists per 1,000 utilizations of CT, classified by municipality type, is shown in Fig 5. The value was highest in metropolis and lowest in town/village. The value in metropolis has decreased by 18.3%, while that in town/village has decreased by 29.9%. The gap in the value between metropolis and town/village has widened from 1.9 to 2.2 times.

**Fig 6:**
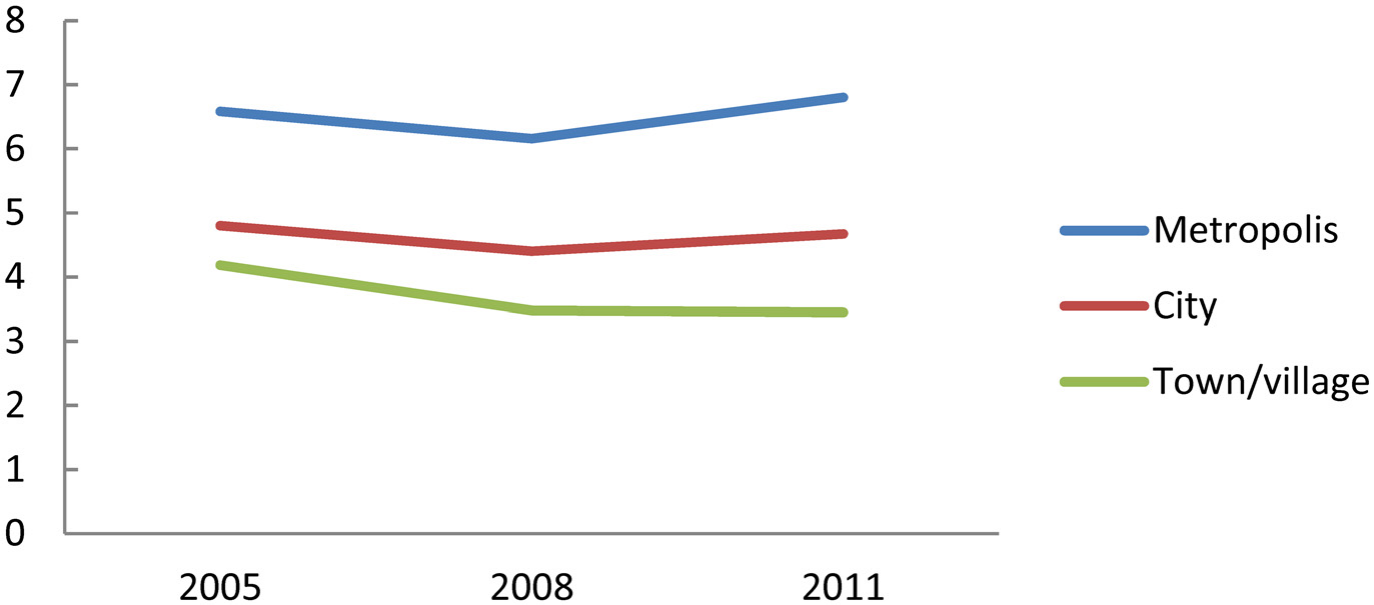
Number of radiologists per 1,000 MRI utilizations, classified by municipality type.

The number of radiologists per 1,000 utilizations of MRI, classified by municipality type, is shown in Fig 6. This value, too, was highest in metropolis and lowest in town/village. The value in metropolis has increased by 3.3%, while that in town/village has decreased by 17.5%. The gap in the value between metropolis and town/village has widened from 1.6 to 2.0 times.

**Fig 7:**
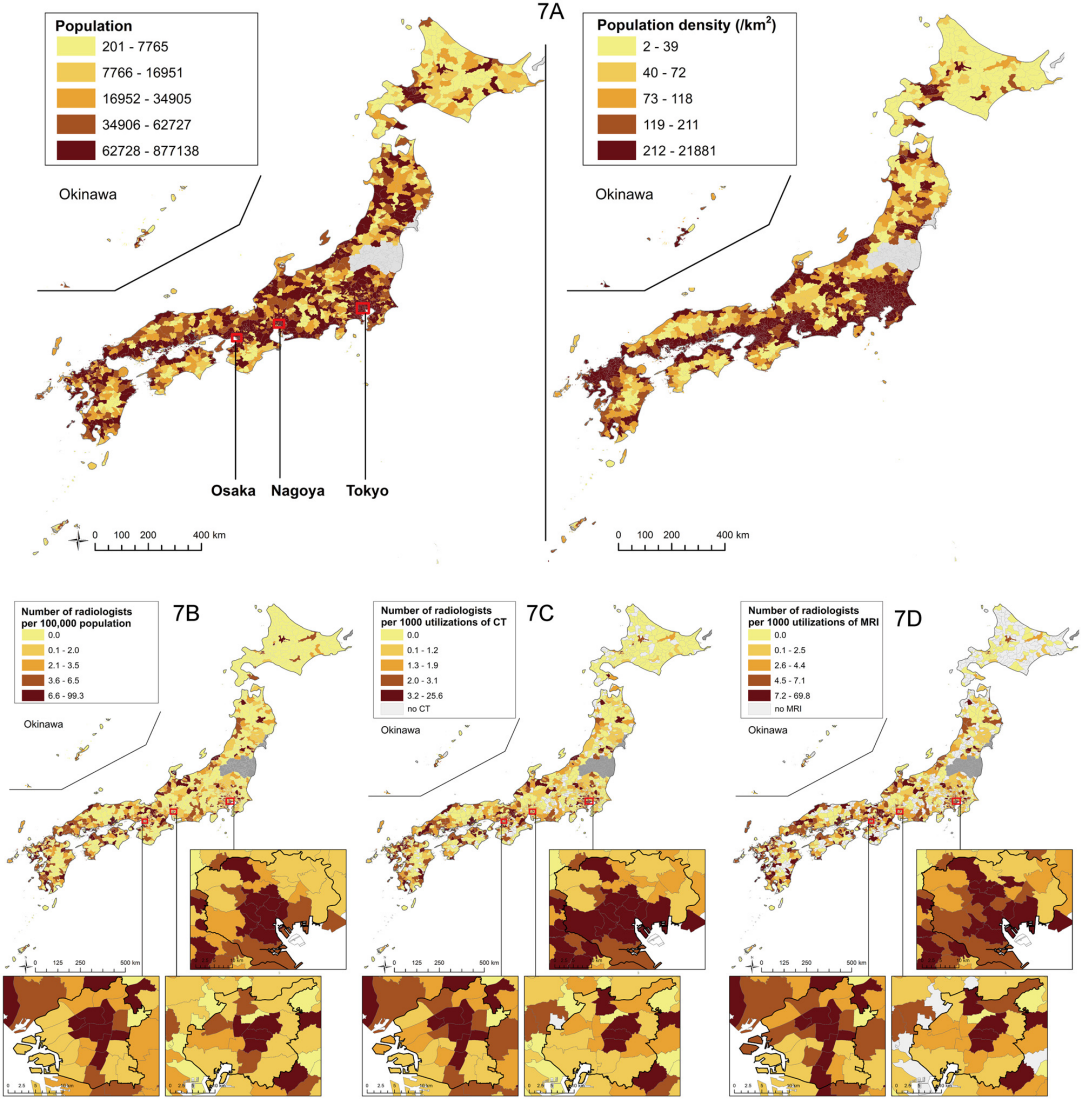
Population, population density (7A), number of radiologists per 100,000 population (7B), number of radiologists per 1,000 CT utilizations (7C), and number of radiologists per 1,000 MRI utilizations (7D) in each municipality in 2011. Quintile points of all values were used as cut-offs for color change.

A color map of the number of radiologists per 100,000 population in each municipality is shown in Fig 7B. For comparison, maps of population and population density are shown in Fig 7A. Radiologists were concentrated in areas with larger population or greater population density. Maps of the number of radiologists per 1,000 CT utilizations (Fig 7C) and that per 1,000 MRI utilizations (Fig 7D) are also shown. These values appear to be higher in areas with larger population or greater population density.

The Spearman’s rank correlation coefficient among municipalities between total population and the number radiologists per 100,000 population was 0.663 (n = 1811, p<0.01), that between total population and the number radiologists per 1,000 CT utilizations was 0.621 (n = 1520, p<0.01), that between total population and the number of radiologists per 1,000 MRI utilizations was 0.477 (n = 1174, p<0.01) (data not shown in tables or figures). These numbers indicate that radiologists are concentrated in municipalities with larger populations.

**Fig 8:**
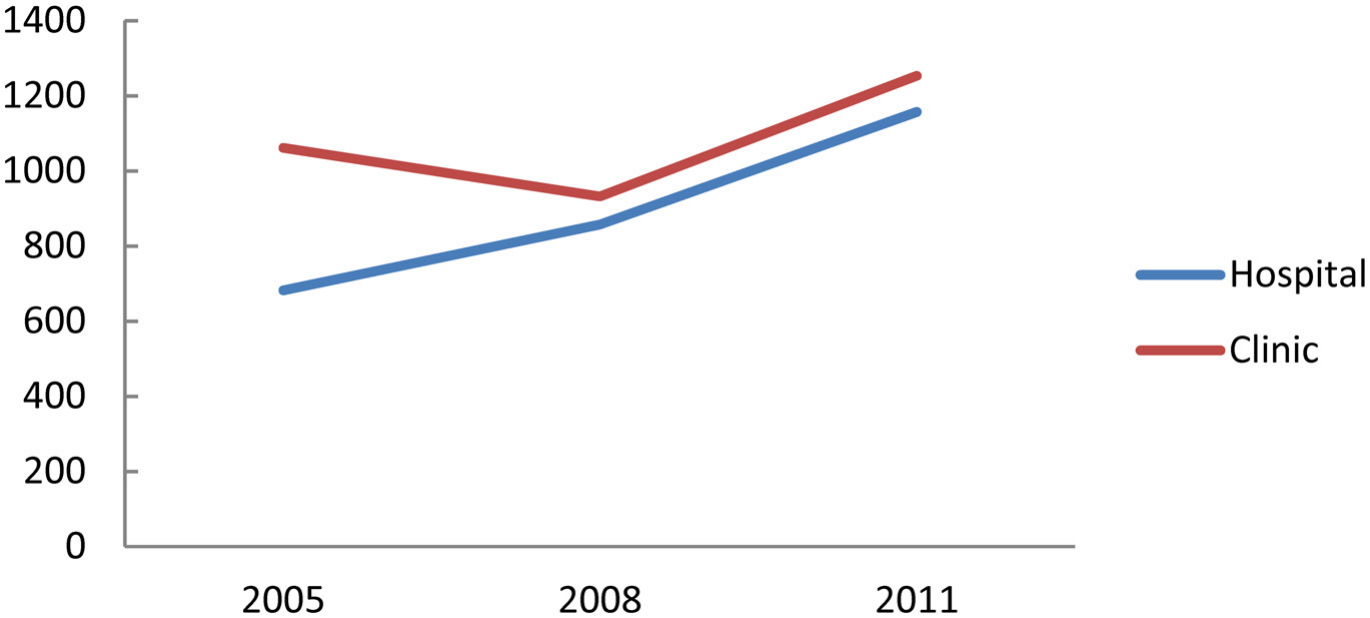
Number of institutions using teleradiology.

**Fig 9:**
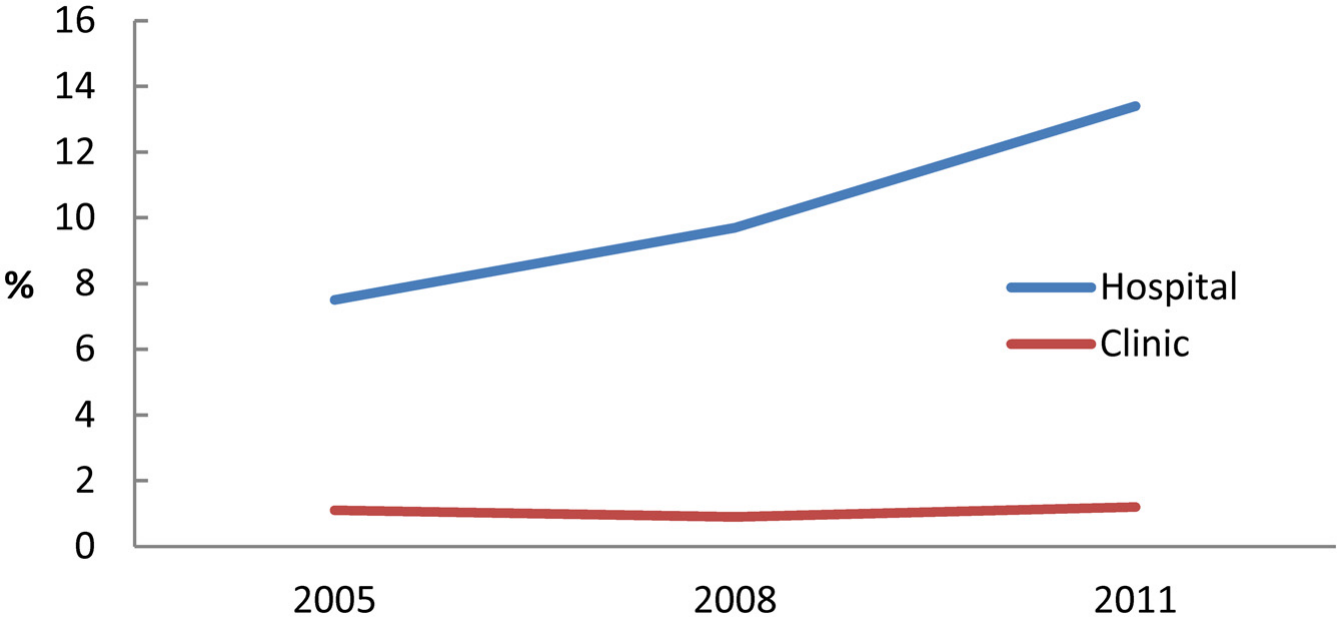
Proportion of institutions using teleradiology.

The number of hospitals and clinics that use teleradiology is shown in Fig 8. The value has increased both for hospitals (increase rate 69.6%) and clinics (18.1%). The proportion of hospitals and clinics using telemedicine is shown in Fig 9. The proportion has increased rapidly (by 78.7%) among hospitals, but it has not substantially increased among clinics (only by 9.1%).

Results of multivariate analysis are shown in Table 2 (hospital) and Table 3 (clinic). Hospitals located in a town/village or city, public hospitals, hospitals with CT/MRI/PET and hospitals with more beds were more likely to use teleradiology than were their counterparts. In contrast the location of clinics was not associated with teleradiology utilization.

**Table 2:** Hospital characteristics associated with using teleradiology (n = 8,632).

	Odds ratio	95% CI	P
Place			
Metropolis	1		
City	1.319	1.11–1.566	0.002
Town/village	1.612	1.257–2.068	<0.001
Ownership			
Private	1		
Public	1.916	1.635–2.246	<0.001
Diagnostic device			
without CT or MRI or PET	1		
with CT or MRI or PET	16.671	10.668–26.052	<0.001
Number of radiologists	1.007	0.983–1.033	0.561
Number of beds (per 100 beds)	1.071	1.024–1.121	0.003

CI: confidence interval

**Table 3:** Clinic characteristics associated with using teleradiology (n = 101,083).

	Odds ratio	95% CI	P
Place			
Metropolis	1		
City	0.764	0.672–0.87	<0.001
Town/village	0.908	0.733–1.125	0.376
Ownership			
Private	1		
Public	3.185	2.58–3.933	<0.001
Diagnostic device			
without CT or MRI or PET	1		
with CT or MRI or PET	18.777	16.721–21.086	<0.001

CI: confidence interval

## Discussion

The results of this study show that a rapid increase in the number of radiologists nationwide has not improved the equity in their geographic distribution. This is quite contrary to the distribution of CT, MRI and PET, which have become more equitable with the increase in their number [2]. In other words, the distribution of radiologists has not followed the spatial competition model derived from market forces, which the distributions of diagnostic devices have followed. The increase in the number of utilizations of diagnostic devices has outpaced the increase in the number of radiologists, which suggests an increasing workload for each radiologist. This is particularly true in rural areas, and the urban-rural gap of the workload seems to have widened. The use of teleradiology, which compensates for the disparity in the number of urban and rural-based radiologists, has increased, particularly among hospitals. Rural hospitals were more likely to use teleradiology than their urban counterparts.

Despite Japan’s much lower reimbursement for imaging than other developed countries, the density of CT and MRI in Japan is the world’s highest. This is partly because the cost of these imaging devices is substantially lower in Japan than in other countries due to the competition among the large domestic manufacturers [22]. The gap has created a severe shortage of radiologists. The proportion of CT and MRI images read by radiologists was only 40% in Japan, while almost all the images were reported by radiologists in 13 of the 14 reported European countries [5]. This means the quality of imaging diagnosis in Japan is not well-controlled.

The numbers of radiologists per 1,000 utilizations of CT and MRI (Figs 4–6) have slightly increased between 2008 and 2011, though they had rapidly decreased between 2006 and 2008. In the 2008 revision of medical service fees by the government, more than 80% of CT and MRI images needed to be interpreted in a day by full-time radiologists of the institution; this became a new requirement for making an insurance claim of interpretation fee for these images. This change has potentially slowed the increased rate of CT and MRI utilizations since 2008, which led to the upward trend between 2008 and 2011 in the Figs.

The real increase in the workload of radiologists would be even larger than what we found in this study. Not only has the use of CT, MRI and PET increased, so have the number of images produced each time these devices are used. This situation would exacerbate the shortage of radiologists [5], which is contrary to the situation in other countries like the United States [23]. In such a shortage, the lopsided distribution of radiologists toward urban areas is unlikely to improve because of the dire need for radiologists even in urban areas. In addition, physicians generally have a strong preference for urban areas and are reluctant to work in rural areas even when offered a financial incentive [24]. These factors would help to explain why spatial competition force did not influence the distribution of radiologists. The discrepancy between the reality and the hypothesis has been observed not only among radiologists, but also among physicians in general [7–9,25,26].

The number of radiologists has and will continue to increase, along with a projected increase in the number of physicians [27]. Also the number of imaging devices will increase because the elderly population and the accompanying medical demand are likewise projected to grow [28]. Considering the number-distribution relationship of radiologists obtained in this study and that of imaging devices in the previous study [2], we can predict that the gap in distribution between radiologists and imaging devices will continue to widen, as radiologists remain concentrated in urban areas while devices continue to diffuse to rural areas. There are many full-time urban radiologists in Japan that regularly work in rural hospitals on a part-time basis. Traditionally, they have filled the urban-rural gap. Recently, however, the rapid increase in their workload makes it increasingly difficult to continue their part-time job. In such a situation, there are three ways to keep this gap from widening: 1) equalize the distribution of radiologists; 2) either stop equalizing device distribution or stop increasing the number of devices; and 3) facilitate the use of teleradiology. In reality, the most feasible option is the third one. A high-speed line that enables transmission of high volume image data is now widely available. In addition, medical records are being shared among hospitals to a greater extent than ever. Thus teleradiology is now a practical way of improving quality of medical care, particularly in areas where there is a shortage of radiologists. As a result, the number of medical institutions using teleradiology is expanding in Japan as in other developed countries [29–31]. This trend should be maintained. However, caution should be taken with the subsequent prevalence of commercial-based teleradiology services whose quality might not meet the required standard.

A policy intervention such as giving financial incentives would be needed to incentivize the appropriate use of teleradiology. The government’s 2014 revision of medical service fees tightened the condition to make an insurance claim of interpretation fee for CT and MRI images [32]. Contrary to the original policy intention to improve the quality of radiology services, this change has the potential to interrupt the current upward trend of teleradiology use, particularly in rural areas, which have to depend on teleradiology to provide radiology services.

The reason that clinics in cities were less likely to use teleradiology services than clinics in metropolis or towns/villages is unknown. It may be that clinics in metropolises have more financial power than those in cities and thus can be afford to use the services. Or it may be that metropolises have more specialised clinics that heavily use CT or MRI, such as comprehensive health check-up clinics and neurosurgery clinics, and thus need to use the services more than cities.

### Limitations

Not all of the radiologists in this study are board-certified. All physicians whose self-reported specialty was radiology were recognized as radiologists in this study. Thus it is possible that our study subjects include trainees and senior radiologists that do not hold the board certificate of the Japan Radiological Society. In 2012, the number of radiologists in this study was 5883, which is substantially more than the 5108 registered board-certified radiologists in that year [33]. Also the radiologists in this study include not only diagnostic but also therapeutic radiologists, though the proportion of therapeutic radiologists among all radiologists is only 16.8% according to the survey of Japanese Board of Medical Specialties [34].

The urban-biased distribution of radiologists might be justified considering the functional difference between urban and rural hospitals. In general, urban hospitals serve as the specialized, technologically-advanced referral institutions compared with rural hospitals that mainly provide primary care. Therefore, urban hospitals need more radiologists than their rural counterparts.

Medical institutions using teleradiology in this study include institutions that both use and provide teleradiology services. Most of the institutions are presumed to be service users, but some large and urban hospitals can be providers. For this reason, the proportion of rural hospitals using teleradiology shown in the results may have been underestimated. In any case, however, most of the teleradiology services in Japan are provided by private companies that were not included in the dataset of this study, and thus the existence of service-provider hospitals would not influence the result.

Not all CT and MRI images were interpreted by radiologists. In Japan it is not unusual for neurologists and neurosurgeons to interpret CT and MRI images of the brain without consultation with radiologists. Thus increase in CT and MRI utilizations does not necessarily mean that the demand for radiologists has increased. Ideally all the images should be read by radiologists, and thus, the rate of increase in the production of images should be met by a corresponding increase in radiologists to interpret them; however, with so many images with such a few radiologists, this is unrealistic. A policy to regulate the number of the imaging devices in each secondary tier of medical care may be a reasonable option.

The geographic unit of analysis is municipality. Municipality is the smallest administrative unit and thus is ideal for this study, which aims at a precise analysis of geographic distribution. However, patients often cross municipality borders to seek medical services, and thus inter-municipality disparity of radiologists and radiology devices is not necessarily a problem. Rather, as reference data for redressing the resource maldistribution, the analysis based on the secondary tier of medical care, which is a complex of several municipalities within which most of the healthcare services are provided and managed, might be better, which should be conducted in the future.

### Conclusions

The rapid increase in the number of radiologists has not equalized their geographic distribution in Japan; in other words, the number-distribution relationship has not followed the market-derived spatial competition force. The result was contrary to the amount-distribution relationship of diagnostic devices such CT and MRI reported previously [2]. The demand-supply balance of radiologists was more disrupted in rural than in urban areas, and the rural-urban gap continues to widen. Teleradiology, which is the only feasible way to close the gap, is spreading; therefore, a policy that supports the increasing use of teleradiology is needed.

## Supporting Information

S1 Fig Lorenz curves of the number of radiologists. (TIF)
