# Supplementary figures and images for: Geographic Distribution of Radiologists and Utilization of Teleradiology in Japan: A Longitudinal Analysis Based on National Census Data
Source: PLoS One. 2015 Sep 30;10(9):e0139723. doi: 10.1371/journal.pone.0139723 (PMC4589480; doi:10.1371/journal.pone.0139723)

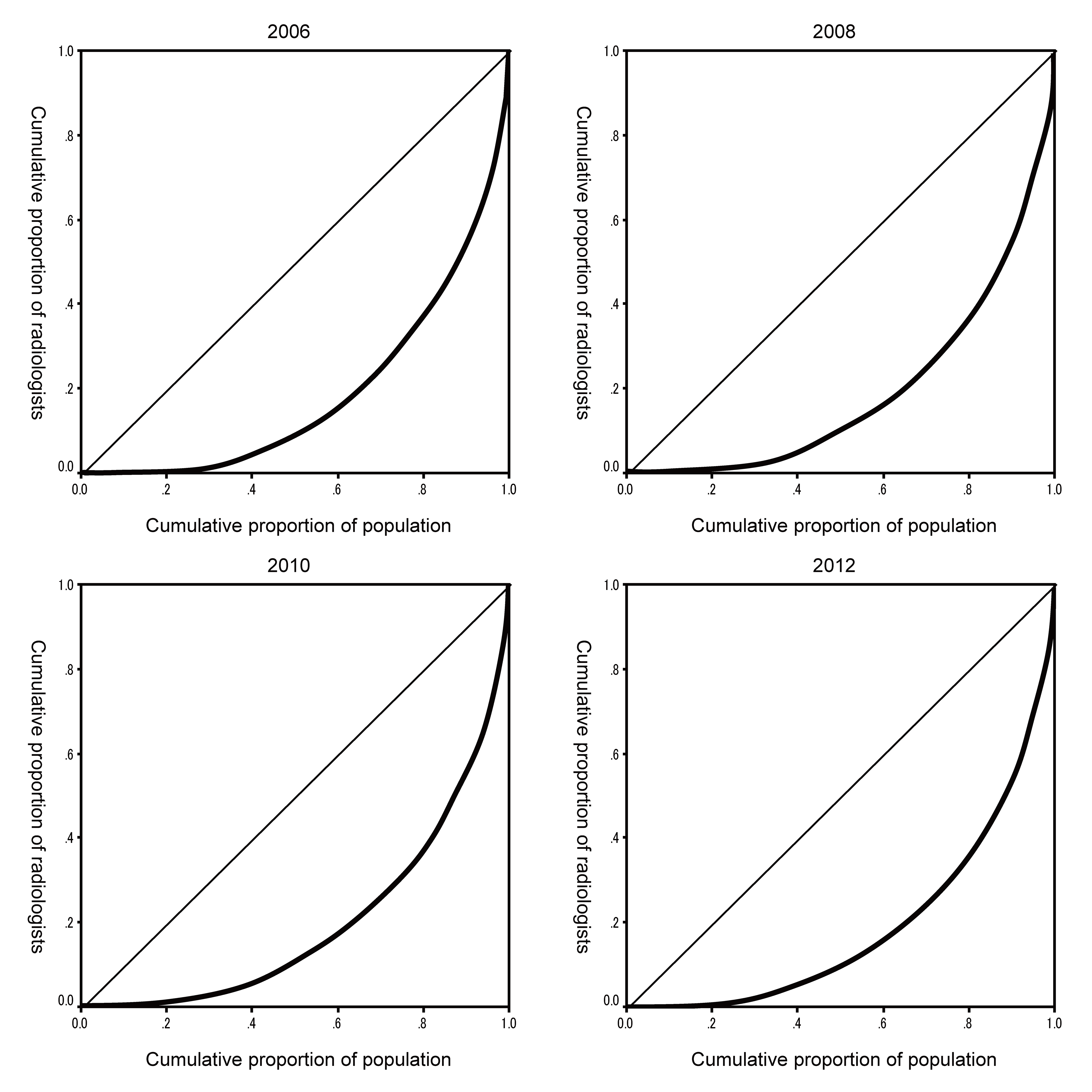

Supplement: S1 Fig — (TIF) [file pone.0139723.s001.tif]
